# A Field Trial to Demonstrate the Potential of a Vitamin B Diet Supplement in Reducing Oxidative Stress and Improving Hygienic and Grooming Behaviors in Honey Bees

**DOI:** 10.3390/insects16010036

**Published:** 2025-01-02

**Authors:** Nemanja M. Jovanovic, Uros Glavinic, Jevrosima Stevanovic, Marko Ristanic, Branislav Vejnovic, Slobodan Dolasevic, Zoran Stanimirovic

**Affiliations:** 1 Department of Parasitology, Faculty of Veterinary Medicine, University of Belgrade, Bul. oslobodjenja 18, 11000 Belgrade, Serbia; nmjovanovic@vet.bg.ac.rs; 2 Department of Biology, Faculty of Veterinary Medicine, University of Belgrade, Bul. oslobodjenja 18, 11000 Belgrade, Serbia; uglavinic@vet.bg.ac.rs (U.G.); mristanic@vet.bg.ac.rs (M.R.); zoran@vet.bg.ac.rs (Z.S.); 3 Department of Economics and Statistics, Faculty of Veterinary Medicine, University of Belgrade, Bul. oslobodjenja 18, 11000 Belgrade, Serbia; branislavv@vet.bg.ac.rs; 4 Institute for Animal Husbandry, Zemun, 11080 Belgrade, Serbia; dolasevicslobodan.izs@gmail.com

**Keywords:** diet supplement, hygienic behavior, honey bee, grooming behavior, oxidative stress

## Abstract

The honey bee is an important insect pollinator that provides critical pollination services for natural and agricultural systems worldwide. However, inadequate food weakens honey bee colonies, making them vulnerable to various biotic and abiotic factors. In this study, we examined the impact of supplementary feeding on bees’ genes for antioxidative enzymes and vitellogenin, oxidative stress parameters, and the hygienic and grooming behavior. The colonies were divided into two experimental groups (with ten hives each): a treatment group that received the plant-based supplement and a control group. The experiment was conducted in two seasons, spring and summer. After the treatment, in both seasons, all the monitored parameters in the treatment group differed from those in the control group. The expression levels of genes for antioxidative enzymes were significantly lower, but the vitellogenin gene transcript level was significantly higher. Values of oxidative stress parameters were significantly lower. The levels of hygienic and grooming behavior were significantly higher. Therefore, our field study indicates that the tested supplement exerted beneficial effects on bees, reflected in reduced oxidative stress and enhanced hygienic and grooming behavior.

## Simple Summary

The nutrition of the honey bee is an important factor essential for maintaining their health. In this study, we examined the impact of supplementary feeding on bees by analyzing the gene expression of antioxidative enzymes and vitellogenin, oxidative stress parameters, and hygienic and grooming behavior. The results of this study indicate that the applied supplement may have a positive effect in reducing oxidative stress and improving hygienic and grooming behavior in honey bees.

## 1. Introduction

The honey bee (*Apis mellifera* L.) is a crucial pollinator in natural and agricultural ecosystems worldwide. Honey bees feed on nectar and pollen, which are vital for brood production, immune system function, oxidative stress resistance, and winter survival. Nectar provides carbohydrates, while pollen is the only source of proteins, lipids, and essential micronutrients [1]. Therefore, diverse flowering plants are necessary to meet the bees’ nutritional requirements [1,2]. Additionally, nectar and pollen must be abundantly available throughout the season to support colony growth and enhance resistance to environmental stressors [3,4,5,6].

Honey bee colony losses are attributed to various stressors, including pathogens, parasites [7,8], and pesticides [9]. However, malnutrition is increasingly recognized as a threat alongside these factors [10,11,12]. Intensive land cultivation reduces floral diversity and lowers the nutritional value of available forage [2,10,13]. Additionally, climate change threatens bee nutrition by altering flowering and the production of nectar and pollen [14,15,16]. Poor nutrition is associated with numerous sublethal effects, including weakened immunity and increased susceptibility to diseases and pesticides [11,17,18,19,20]. To ensure food reserves, mitigate the lack of natural pollen and enhance colony strength before the foraging season, beekeepers often use various substitute diets [21,22,23,24,25]. Different supplements which contain protein-rich ingredients such as soy, peas, yeast, casein, and eggs are often used in beekeeping. Some diets include collected pollen, which has been shown to increase consumption and brood rearing. Different supplements are tested in a laboratory and field conditions for consumption/palatability, colony productivity, pest and disease response, and physiological response [reviewed in 24]. The efficacy of these diets varies depending on their nutritional composition and testing conditions. It can stimulate immune responses, prevent oxidative stress in worker bees, and increase resistance to the microsporidian *Nosema ceranae* [26,27,28,29,30]. Moreover, such feeding can contribute to colony strength, i.e., increase the number of adult bees and brood area [25] and significantly influence bee social immunity [31]. In our earlier research, supplement “B+” showed positive effects under laboratory conditions on the survival of bees infected with the endoparasite *N. ceranae* and on the reduction of oxidative stress [29]. Furthermore, in a field experiment, the mentioned supplement positively influenced the strength parameters of bee colonies and contributed to a reduction in the amount of *N. ceranae* [25].

Social insects such as honey bees have developed various behaviors to prevent the spread of pathogens and parasites within their colonies [32,33,34]. These behaviors involve complex interactions among colony members and adaptive responses that mitigate the impacts of infectious and parasitic diseases on colonies. This collective mechanism is known as “social immunity” [32,35,36,37,38] and includes strategies such as the spatial segregation of high-risk individuals [39,40], contact reduction and the removal of infected bees [41,42,43], and the altruistic self-removal of infected worker bees [44]. Other significant aspects of social immunity include grooming and hygienic behavior [45,46,47,48,49].

Grooming behavior involves activities by which adult bees remove and injure mites from their own body (auto-grooming) or the bodies of other bees (allogrooming) [45]. This behavior significantly contributes to the colony’s resistance against mites by increasing mite mortality and regulating their population growth [49,50,51]. Notably, Africanized bees provide some of the best examples of natural, long-term tolerance to *Varroa* mites in colonies [49,52,53,54].

Hygienic behavior is the ability of worker bees to detect, uncap, and remove diseased or dead brood from cells [33,34]. This behavior serves as an effective defense mechanism against bee diseases such as American foulbrood [34,55,56], chalkbrood [57], and infestations by *Varroa* mites [34,58].

In this study, we investigated the effects of the “B+” supplement on the expression of genes for antioxidant enzymes and the vitellogenin gene, along with its impact on antioxidant enzymes activities in bee colonies. We also examined the supplement’s influence on the hygienic and grooming behaviors of bees.

## 2. Materials and Methods

### 2.1. Experimental Design

**Figure 1:**
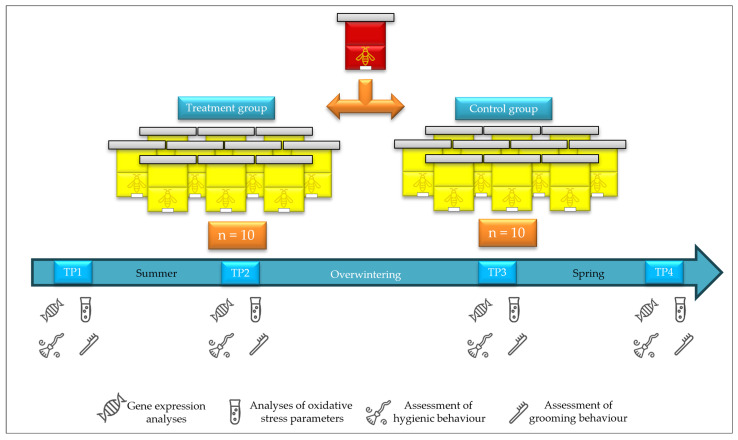
Experimental design. Sampling and evaluations were performed at four time points during the experiment: before (TP1) and after (TP2) the first phase, and before (TP3) and after (TP4) the second phase.

The experimental design was conducted in accordance with the methodology described in Jovanovic et al. [25] and represents a continuation of research on the effects of the supplement “B+”. The experiment was conducted in Podnemic (Ljubovija municipality), a village in the west of Serbia (44° 10′ 17″ N; 19° 25′ 23″ E). The colonies of *Apis mellifera carnica* [59] were kept in Albert-Žnidaršič (AŽ) hives and led by queens originating from the same mother. In total, twenty bee colonies were divided into two experimental groups (treatment and control) of ten colonies each. In the treatment group (TG), colonies were fed with the “B+” supplement, which was prepared according to the manufacturer’s instructions—1 g of the supplement was dissolved in 1 L of 40% *w*/*v* sugar syrup. In the control group (CG), colonies were fed with 40% (*w*/*v*) sugar syrup (Figure 1). Each hive in the experiment represented one experimental unit.

Before the start of the experiment, the colonies were equalized according to colony strength parameters (areas with open and sealed brood, honey and pollen/beebread reserves, and the number of adult bees) following the procedure described by Delaplane et al. [60]. The experiment was conducted in two phases: (i) in the summer during the preparation of the bees for overwintering and (ii) in the spring during the preparation of the colonies for the foraging. Each phase lasted 21 days. During both phases, the supplement solution in the TG and sugar syrup in the CG were applied using Millers feeders (500 mL per colony on a daily basis). From each hive, a total of 70 worker bees (foragers) were sampled for gene expression analyses and to determine oxidative stress parameters. The assessments for hygienic and grooming behaviors were also conducted. Sampling and evaluations took place at four time points: before (TP1) and after the first phase (TP2), and before (TP3) and after the second phase (TP4) of the experiment (Figure 1).

### 2.2. Gene Expression Analyses

Each sample (40 bees) was macerated and homogenized in 6 mL of phosphate-buffered saline (PBS). The total RNA was extracted using the Quick-RNA MiniPrep Kit (Zymo Research, Irvine, CA, USA) following the manufacturer’s instructions. The RNA samples were equalized to contain 1000 ng of RNA. The RNA was immediately converted into complementary DNA (cDNA) using the RevertAid™ First Strand cDNA Synthesis Kit (Thermo Fisher Scientific, Vilnius, Lithuania) according to the manufacturer’s protocol. The cDNA was stored at −20 °C until further analysis.

The expression levels were measured for genes involved in antioxidative protection (Cu/Zn superoxide dismutase, Mn superoxide dismutase, catalase, and glutathione S-transferase) and for vitellogenin, a gene related to nutrition and development. Quantitative real-time PCR (qPCR) was performed using the SYBR Green technique in Rotor-Gene Q 5plex real-time PCR system (Qiagen, Hilden, Germany). Each qPCR reaction was conducted in a final volume of 20 µL, containing 10 µL of FastGene IC Green 2 × qPCR Universal Mix (KAPA Biosystems, Wilmington, MA, USA), 0.8 µL (50 nM) of each primer (forward and reverse), 7.4 µL of dH_2_O, and 1 µL of cDNA template. The cycling parameters consisted of an initial denaturation step at 95 °C for 2 min, followed by 45 cycles of denaturation at 95 °C for 5 s, annealing at 60 °C for 30 s, and elongation at 72 °C for 5 s. Primers are listed in Table 1. Gene expression levels were quantified using the 2^−ΔCt^ method, with the housekeeping gene β-actin used as the internal control for normalization [61].

### 2.3. Determination of Oxidative Stress Parameters

To evaluate the activities of superoxide dismutase (SOD), catalase (CAT), and glutathione S-transferase (GST), and to measure the concentration of malondialdehyde (MDA), a pooled sample of 30 bees from each hive was used. Briefly, the bees were ground into a fine powder with liquid nitrogen using a mortar and pestle and then homogenized with Tris-HCl buffer, pH 7.4. The crude homogenates were centrifuged at 10,000× *g* for 10 min at 4 °C. The supernatant was aliquoted and stored at −20 °C for further analysis. Enzyme activities of SOD, CAT, and GST were assessed according to the methods described by Misra and Fridovich [64], Aebi [65], and Habig et al. [66], respectively, while the concentration of MDA was determined following the method of Girotti et al. [67]. Protein concentrations in the analyzed samples were quantified using the Bradford method. Analyses were performed using a UV/VIS spectrophotometer BK-36 S390 (Biobase, Jinan, China). Each measurement was conducted in triplicate.

### 2.4. Assessment of Hygienic and Grooming Behavior

The hygienic behavior was evaluated using the “pin-killed” brood assay described in Stanimirović et al. [68]. Briefly, one frame from each colony containing uniform capped brood was selected. On each frame, a 6 × 5 cm section of capped brood cells was perforated using an entomological pin to kill the brood. The frames were marked and reintroduced into the colony. After 24 h, the number of cleaned cells was counted. The level of hygienic behavior was expressed as the percentage of cleaned cells.

Grooming behavior was evaluated by the methodology described in Stevanović et al. [69]. At four time points, at least 30 mites were sampled from the anti-*Varroa* bottom board [70]. Mites were collected with a fine brush to prevent additional damage. Only adult mites were used to examine grooming behavior. Mites lighter than ochre brown were classified as immature, while darker ones were considered adults. All types of injuries were considered, except for regular dorsal dimples—one or two depressions or hollows in predictable locations on the dorsal idiosoma as recommended by Davis [71]. The level of grooming behavior was expressed as the percentage of damaged mites.

### 2.5. Statistical Analysis

Data for gene expression (GST, MnSOD, CuZnSOD, CAT, and vitellogenin), oxidative stress parameters (SOD, CAT, GST, and MDA), and hygienic and grooming behaviors were tested for normality using the Shapiro–Wilk test. Since the gene expression data were not normally distributed (Shapiro–Wilk test, *p* < 0.05), a log_10_ (y + 2) transformation was applied. Groups were compared using two-way ANOVA with repeated measures in one factor, followed by Tukey’s test for within-group comparisons and Sidak’s test for between-group comparisons over time. Significant differences were considered at *p* < 0.05, *p* < 0.01, and *p* < 0.001 significance levels. Statistical analyses were performed using GraphPad Prism 7 software (GraphPad, San Diego, CA, USA).

## 3. Results

### 3.1. Comparison Between Groups

For analyzing gene expression level data, oxidative stress parameters, and hygienic and grooming behavior between the two experimental groups, comparisons were considered only if the group × time interaction showed statistical significance (*p* < 0.05). In this experiment, interactions, group × time, were statistically significant (*p* < 0.05) for all the mentioned parameters (Supplementary Table S1).

#### 3.1.1. Gene Expression

**Figure 2:**
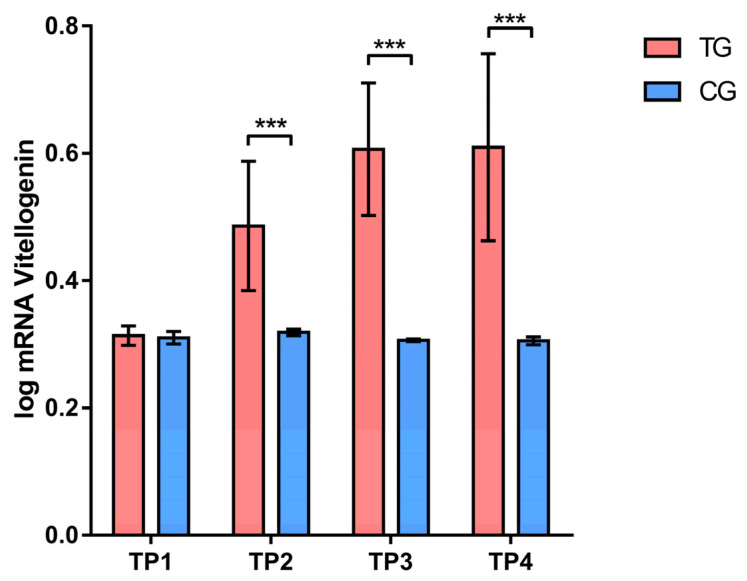
Comparison of expression levels of vitellogenin in different sampling occasions between treatment and control group. *** *p* < 0.001; TP1—summer before treatment; TP2—summer after treatment; TP3—spring before treatment; TP4—spring after treatment; TG—Treatment group; CG—Control group. Error bar stands for standard deviation.

**Figure 3:**
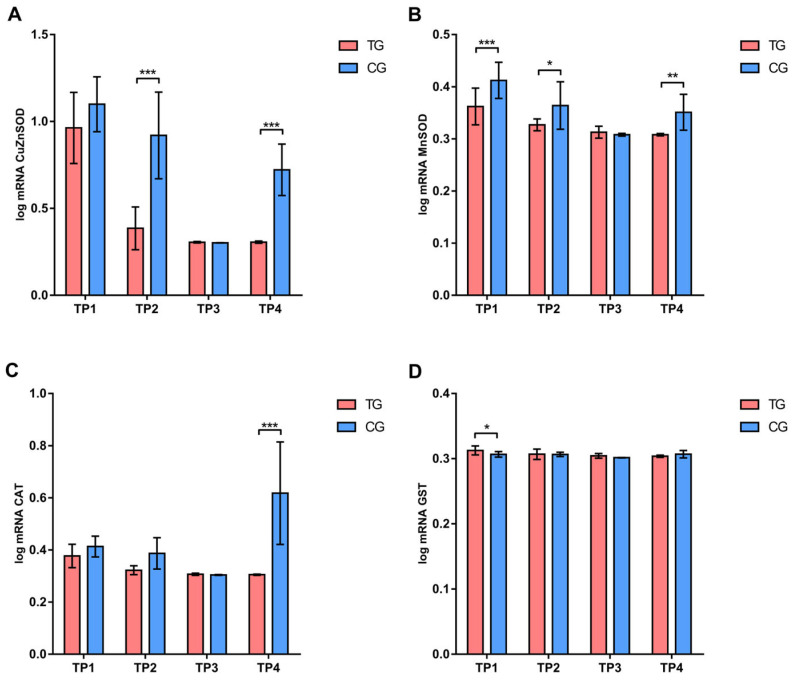
Comparison of expression levels of (**A**) CuZn superoxide dismutase, (**B**) Mn superoxide dismutase, (**C**) catalase, and (**D**) glutathione S-transferase in different sampling occasions between treatment and control group. * *p* < 0.05; ** *p* < 0.01; *** *p* < 0.001; TP1—summer before treatment; TP2—summer after treatment; TP3—spring before treatment; TP4—spring after treatment; TG—Treatment group; CG—Control group. Error bar stands for standard deviation.

According to Sidak’s test, the expression of the *vitellogenin* gene was significantly higher (*p* < 0.001) in the treatment group at the end of the first phase (TP2), before (TP3) and at the end of the second phase (TP4) (Figure 2). A significantly lower (*p* < 0.001) expression of CuZnSOD gene was recorded in the treatment group compared to the control group at the TP2 and TP4 measurement points. Additionally, the expression levels of MnSOD genes (at TP1, TP2, and TP4) and CAT gene (at TP4) were significantly lower (*p* < 0.05, *p* < 0.01, and *p* < 0.001) in the treatment group compared to the control group. On the contrary, a significantly higher (*p* < 0.05) level of GST gene expression was affirmed in the treatment group compared to the control group only at TP1 (Figure 3).

#### 3.1.2. Oxidative Stress Parameters

**Figure 4:**
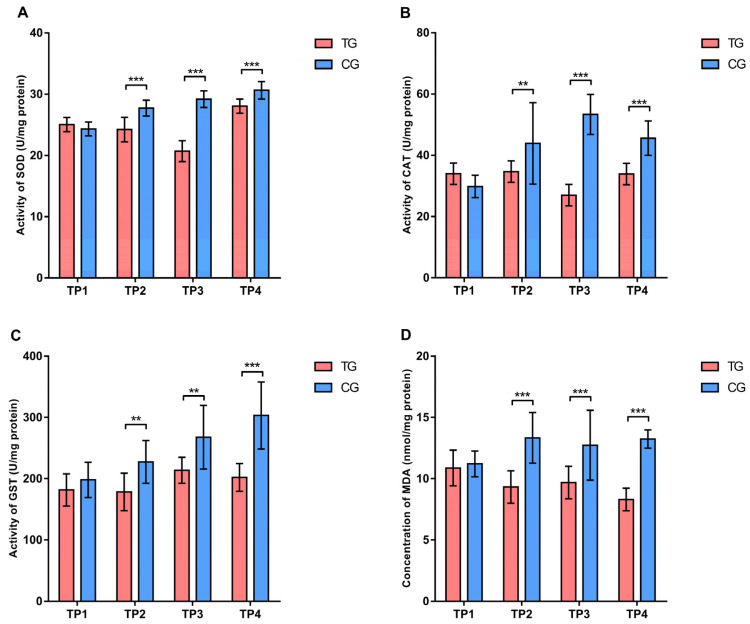
Comparison of activity of (**A**) superoxide dismutase, (**B**) catalase, (**C**) glutathione S-transferase, and (**D**) concentration of malonyl-dialdehyde in different sampling occasions between treatment and control group. ** *p* < 0.01; *** *p* < 0.001; TP1—summer before treatment; TP2—summer after treatment; TP3—spring before treatment; TP4—spring after treatment; TG—Treatment group; CG—Control group. Error bar stands for standard deviation.

Analyzing the activity of SOD, CAT, and GST enzymes using Sidak’s test, significantly lower values (*p* < 0.01 and *p* < 0.001) were observed in the treatment group compared to the control group at the TP2, TP3, and TP4 measurement points (Figure 4). Additionally, the concentration of MDA was also significantly lower (*p* < 0.001) in the treatment group compared to the control group at TP2, TP3, and TP4 (Figure 4).

#### 3.1.3. Hygienic and Grooming Behavior

**Figure 5:**
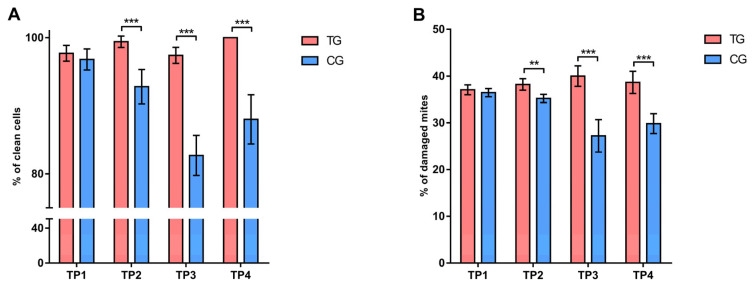
Comparison of (**A**) hygienic and (**B**) grooming behavior in different occasions between treatment and control group. ** *p* < 0.01; *** *p* < 0.001; TP1—summer before treatment; TP2—summer after treatment; TP3—spring before treatment; TP4—spring after treatment; TG—Treatment group; CG—Control group. Error bar stands for standard deviation.

Analyzing the data on hygienic behavior using the Sidak test, a significantly higher (*p* < 0.001) level was confirmed in the treatment group compared to the control group at the TP2, TP3, and TP4 time points (Figure 5). Similarly, values of grooming behavior were also significantly higher (*p* < 0.01 and *p* < 0.001) in the treatment group compared to the control group at TP2, TP3, and TP4 (Figure 5).

### 3.2. Comparison Within Groups

#### 3.2.1. Gene Expression

The expression of the vitellogenin gene in the treatment group was highest at TP4, while it remained unchanged in the control group (Supplementary Figure S1). Based on the results of the Tukey’s test within the treatment group, significantly higher (*p* < 0.001) expression levels of genes for CuZnSOD, MnSOD, and GST were recorded at the beginning of the experiment (TP1) compared to other time points. In the control group, similar results were confirmed for CuZnSOD and MnSOD, while the gene expression for CAT was highest (*p* < 0.001) at TP4 (Supplementary Figure S2).

#### 3.2.2. Oxidative Stress Parameters

According to Tukey’s test, the most significant changes related to the activity of antioxidant enzymes in the treatment group were observed for SOD enzymes, with the significantly highest (*p* < 0.01) activity recorded at TP4. In the control group, CAT activity values were highest at TP3, while SOD and GST activities peaked at TP4. The concentration of MDA in the treatment group was highest at the beginning of the experiment (TP1). In contrast, in the control group, the values were increased at TP2, TP3, and TP4 compared to the beginning (Supplementary Figure S3).

#### 3.2.3. Hygienic and Grooming Behavior

The significantly highest (*p* < 0.01, *p* < 0.001) level of hygienic behavior in bees within the treatment group was confirmed at TP4. In contrast, a decrease in the expression of hygienic behavior was recorded in the control group. Similar results were obtained in the analysis of grooming behavior (Supplementary Figure S4).

## 4. Discussion

Despite the widespread use of nutrition supplements, the benefits of feeding artificial diets vary in large-scale beekeeping operations. This study tested the effects of the plant-based supplement “B+” on gene expressions related to antioxidant enzymes and vitellogenin, oxidative stress parameters, and its impact on hygienic and grooming behaviors.

We examined the expression of the vitellogenin gene, a key molecular marker for assessing colony nutritional status. In this experiment, colonies fed the supplement “B+” showed significantly higher vitellogenin gene expression levels than the control group. The treatment group exhibited elevated vitellogenin expression at all analyzed time points except TP1. In our previous laboratory experiment, the tested supplement “B+” positively affected vitellogenin gene expression, with the highest levels observed in the treatment group [29]. On the contrary, vitellogenin gene expression did not change during the experiment in control group, which received sugar syrup. These results are in line with studies where it was found that vitellogenin expression is linked to diet quality [10,26,29,72,73,74,75,76,77,78,79]. The connection between diet and vitellogenin expression has also been studied in other laboratory and hive experiments. Most studies have focused on adding pollen to the diet and its effect on vitellogenin expression. Interestingly, feeding bees with *Erica* spp. pollen, which has a high lipid content, had the most significant impact on vitellogenin expression [5]. Additionally, colonies with access to diverse forage showed higher vitellogenin expression [78]. Our results are in line with those of Alaux et al. [75], which were obtained from colonies in landscapes enriched with melliferous catch crops and surrounded by semi-natural habitats. These colonies showed improved bee physiology, specifically in terms of fat body mass and vitellogenin levels. In laboratory experiments, the supplement “Beewell AminoPlus” positively affected vitellogenin expression even in the presence of *N. ceranae* infection [26]. Moreover, water extracts from the mushrooms *Agaricus blazei* and *A. bisporus* have been shown to provide protective effects on bees and, along with their immunostimulatory properties, positively impact vitellogenin levels [27,28]. In summary, adequate nutrition enhances vitellogenin expression in bees, boosting immune responses and improving colony performance, as evidenced by positive correlations with brood quantity, adult bee mass, and food reserves in the hive [25,26,27,28,78,79].

The antioxidant response is a crucial factor in bee health. Bee pathogens and parasites are significant biotic factors that can disrupt this response [25,27,28,29,61]. The antioxidant response is also linked to bee habitat [80], while differences were also observed between summer and winter bees [81]. In our experiment, we noted differences in gene expression for antioxidant enzymes between the two experimental groups at most analyzed time points. Overall, the expression levels of the CuZnSOD and MnSOD genes were lower in the treatment group compared to the control group. Expression levels of these genes decreased at the end of the first phase (TP2). By the end of the experiment (TP4), expression levels increased in the control group but remained unchanged in the treatment group. Additionally, differences in SOD enzyme activity were observed both between and within the experimental groups. However, at some time points we found that gene expression did not always correlate with enzyme activity. This observation aligns with previous studies, which confirm that gene expression does not always correlate with enzyme activity due to additional regulatory mechanisms involved in the antioxidant response [81,82,83]. Similar findings were recorded for CAT gene expression, with lower values in the treatment group compared to the control group only at the end of the experiment (TP4). Analysis of CAT enzyme activity showed a different trend, with lower values in the treatment group at TP2, TP3, and TP4. Changes in GST gene expression were evident only at the beginning of the experiment, when levels were higher in the treatment group than in the control group. However, biochemical analysis confirmed lower GST enzyme activity in the treatment group throughout the experiment. These differences in GST activity suggest that bees fed with this supplement may have an altered detoxification capacity [84,85]. The differences observed in this experiment may be linked to the type of diet received by the two experimental groups. In previous studies [25,86,87], it was observed that a diet based on sugar syrup, such as that provided to the control group (CG), can promote the growth of the microsporidium *N. ceranae*, which has been shown to induce oxidative stress [29]. In contrast, the lower gene expression and enzyme activity levels observed in the treatment group (TG) could be attributed to the antioxidant properties of certain substances in the “B+” supplement, which neutralize reactive oxygen species (ROS). Antioxidants mitigate ROS—the primary agents that stimulate the expression of antioxidant enzyme genes—which may lower the expression levels recorded in the treatment group. In this research, a lower concentration of MDA was observed. These results are consistent with our previous study [29], where bees fed with this supplement had a lower concentration of MDA, even when infected with *N. ceranae*.

Additionally, this study examined parameters of social immunity in bees, specifically hygienic and grooming behaviors. These heritable behaviors significantly influence a colony’s resistance to pathogens and parasites [68,88]. In our research, hygienic behavior was expressed more in the treatment group colonies compared to the control ones. A higher level of hygienic behavior was observed by the end of the first phase (TP2) and peaked at the end of the experiment (TP4). Similarly, the positive effect of the BeeWell AminoPlus supplement on hygienic behavior was found by Stanimirovic et al. [31]. In a comprehensive study involving experimental groups treated with the supplement and infected with viruses and/or *N. ceranae*, the results showed that BeeWell AminoPlus significantly stimulated hygienic behavior. The control group showed the highest hygienic behavior level at the beginning (TP1), with lower levels recorded at subsequent time points. Similarly, in the study of Stanimirovic et al. [31], a continuous decline in hygienic behavior was recorded in the non-supplemented group. In other studies, the availability or lack of sucrose syrup and brood manipulation did not significantly alter this behavior. However, some alternative treatments against *Varroa* and/or *Nosema* have been found to affect the bees’ hygienic behavior. For instance, thymol increased cell opening and the removal of dead brood, indicating a stimulative effect on hygienic behavior [89]. Conversely, migratory beekeeping practices [90] and the pesticide imidacloprid [91] negatively impact hygienic behavior.

Grooming behavior is affected by numerous biological and environmental factors [49,69,92,93,94,95,96]. For example, grooming behavior can also be artificially stimulated by dusting the colony with powdered sugar [69]. Indeed, the expression of grooming behavior was higher in the supplemented group than in the control group, with more grooming observed during the second phase. In contrast, grooming in the control group showed a declining trend. In a previous study by Jovanovic et al. [29], the tested supplement (“B+”) did not affect *Varroa* infestation rates. However, one of the observations in the same study was a difference in the number of worker bees in the treatment group, which also suggests a higher overall number of *Varroa* mites. This indicates that the expression of grooming behavior in the treatment group may have been influenced by the tested supplement. Currently, the effect of diet on grooming behavior expression has not been investigated, and it is necessary to identify genes closely associated with this behavior. In a recent study by Russo et al. [97], the expression levels of eleven candidate genes thought to be involved in grooming and hygienic behaviors in adult worker bees were analyzed. The results indicated that some of the analyzed genes (*Nrx1*, *Oa1*, *Obp4*, *Obp14*, *Obp16*, *Obp18*, *Spf45*, and *CYP9Q3*) are part of a specific response to *Varroa* mite infestation. Hygienic and grooming behaviors are polygenic traits in bees [98,99,100,101,102,103,104,105], so there is great possibility of micro- and macronutrients and other external factors to influence their expression, as already shown in studies of Stanimirovic et al. [31], Colin et al. [90] and Wu-Smart and Spivak [91].

## 5. Conclusions

This study demonstrated that the tested supplement positively influenced honey bee health. Compared to the control, the group fed the B+ supplement exhibited higher vitellogenin gene expression levels, along with lower expression levels of antioxidant genes and reduced activities of antioxidant enzymes. Additionally, supplemental feeding showed beneficial effects on hygienic and grooming behaviors. These findings are significant as they suggest that the tested dietary supplement can enhance key physiological and behavioral traits in honey bees, potentially improving colony resistance.

## Figures and Tables

**Table 1:** Primers used for real-time polymerase chain reaction.

Primer	Sequence 5′–3′	Reference
Cu/ZnSOD-F	TCAACTTCAAGGACCACATAGTG	[62]
Cu/ZnSOD-R	ATAACACCACAAGCAAGACGAG	
MnSOD-F	GTCGCCAAAGGTGATGTCAATAC	[62]
MnSOD-R	CGTCTGGTTTACCGCCATTTG	
GST-F	AGGAGAGGTGTGGAGAGATAGTG	[62]
GST-R	CGCAAATGGTCGTGTGGATG	
CAT-F	TTCTACTGTGGGTGGCGAAAG	[62]
CAT-R	GTGTGTTGTTACCGACCAAATCC	
VgMC-F	AGTTCCGACCGACGACGA	[63]
VgMC-R	TTCCCTCCCACGGAGTCC	
β-actin-F	TTGTATGCCAACACTGTCCTTT	[63]
β-actin-R	TGGCGCGATGATCTTAATTT	

F—forward; R—reverse; GST—glutathione S-transferase; Cu/ZnSOD—cytoplasmic Cu-Zn superoxide dismutase; MnSOD—mitochondrial Mn superoxide dismutase; CAT—catalase; VgMC—vitellogenin; β-actin—beta actin.

## Data Availability

The data presented in this study are available on request from the corresponding author.

## Supplementary Materials

The following supporting information can be downloaded at: https://www.mdpi.com/article/10.3390/insects16010036/s1, Table S1: ANOVA interactions; Figure S1: Comparison of expression levels of vitellogenin in different sampling occasions within treatment and control group; Figure S2: Comparison of expression levels of (A) CuZn superoxide dismutase, (B) Mn superoxide dismutase, (C) catalase, (D) glutathione S-transferase in different sampling occasions within treatment and control group; Figure S3: Comparison of activity of (A) superoxide dismutase, (B) catalase, (C) glutathione S-transferase and (D) concentration of malonyl-dialdehyde in different sampling occasions within treatment and control group; Figure S4: Comparison of (A) hygienic and (B) grooming behavior in different occasions within treatment and control group.
