# Supplementary material for: A Field Trial to Demonstrate the Potential of a Vitamin B Diet Supplement in Reducing Oxidative Stress and Improving Hygienic and Grooming Behaviors in Honey Bees
Source: Insects. 2025 Jan 2;16(1):36. doi: 10.3390/insects16010036 (PMC11765757; doi:10.3390/insects16010036)
Supplement: Supplementary file 1 [file insects-16-00036-s001.zip › insects-3321374-supplementary.pdf]

Supplementary Table S1. ANOVA interactions

	Parameters	Variations	p
Gene expression analyses	CuZn superoxide dismutase	Interaction	<0.0001
		Time	<0.0001
		Group	<0.0001
	MnSOD superoxide dismutase	Interaction	0.0067
		Time	<0.0001
		Group	0.0003
	Catalase	Interaction	<0.0001
		Time	<0.0001
		Group	<0.0001
	Glutathione S-transferase	Interaction	0.0231
		Time	0.0005
		Group	0.1975
	Vitellogenin	Interaction	<0.0001
		Time	<0.0001
		Group	<0.0001
Oxidative stress parametres	Superoxide dismutase	Interaction	<0.0001
		Time	<0.0001
		Group	<0.0001
	Catalase	Interaction	<0.0001
		Time	<0.0001
		Group	<0.0001
	Glutathione S-transferase	Interaction	0.0005
		Time	<0.0001
		Group	<0.0001
	Malonyl Dialdehyde	Interaction	<0.0001
		Time	0.5251
		Group	<0.0001
Social immunity parameters	Hygienic behavior	Interaction	<0.0001
		Time	<0.0001
		Group	<0.0001
	Grooming behavior	Interaction	<0.0001
		Time	<0.0001
		Group	<0.0001

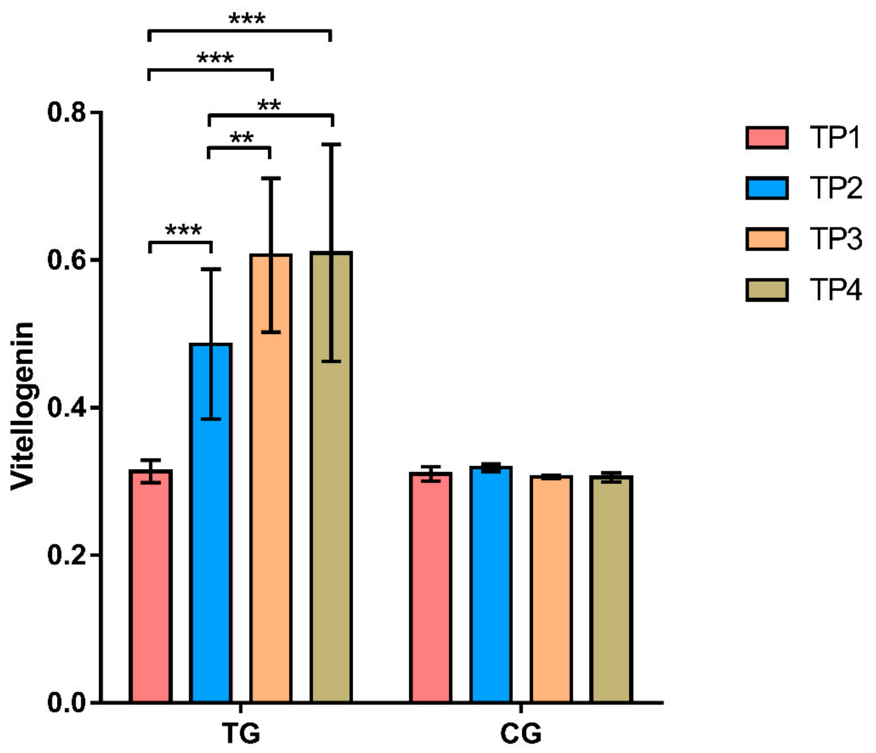

Supplementary Figure S1. Comparison of expression levels of vitellogenin in different sampling occasions within treatment and control group. ****p*<0.001; TP1 - summer before treatment; TP2 - summer after treatment; TP3 - spring before treatment; TP4 - spring after treatment. TG - Treatment group; CG – Control group.

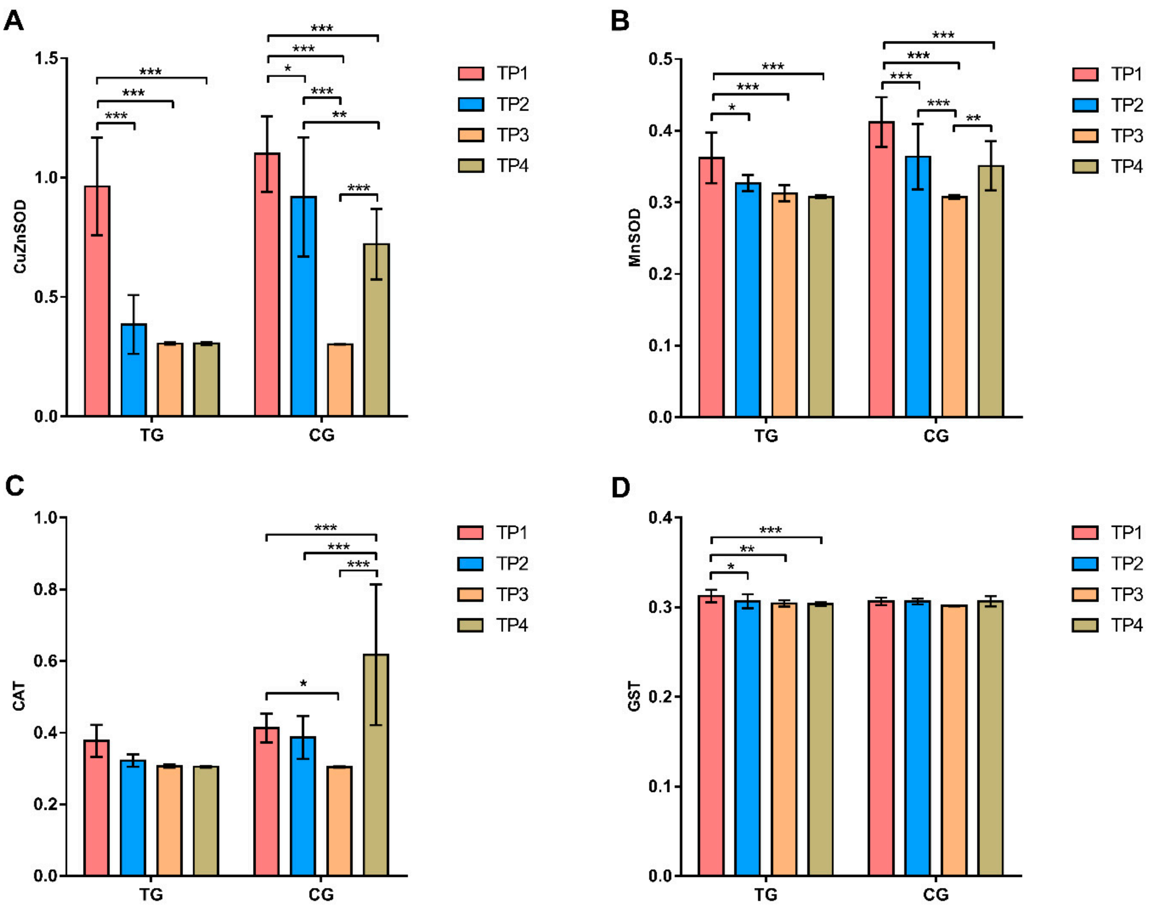

Supplementary Figure S2. Comparison of expression levels of (**A**) CuZn superoxide dismutase, (**B**) Mn superoxide dismutase, (**C**) catalase, (**D**) glutathione S-transferase in different sampling occasions within treatment and control group. **p*<0.05; ***p*<0.01; ****p*<0.001; TP1 - summer before treatment; TP2 - summer after treatment; TP3 - spring before treatment; TP4 - spring after treatment. TG - Treatment group; CG – Control group.

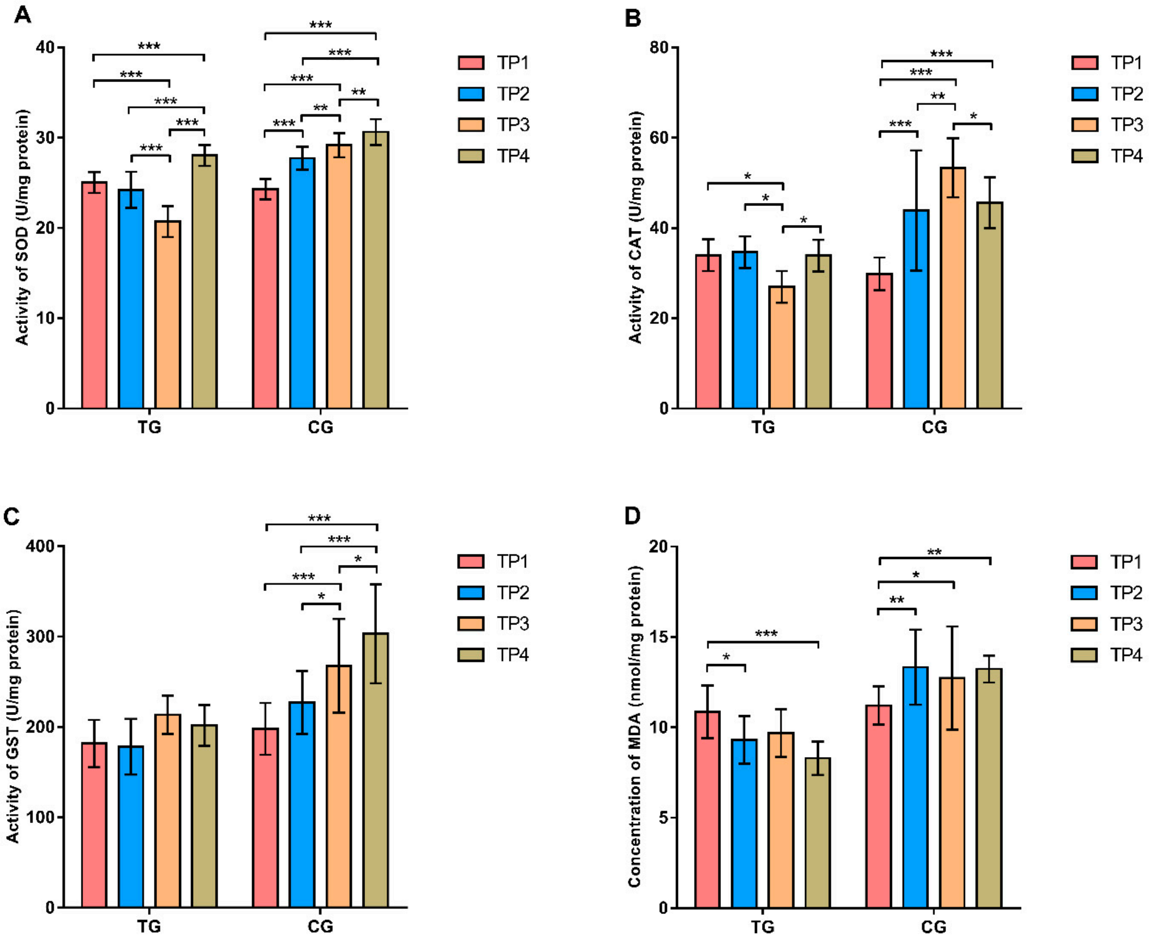

Supplementary Figure S3. Comparison of activity of (**A**) superoxide dismutase, (**B**) catalase, (**C**) glutathione S-transferase and (**D**) concentration of malonyl-dialdehyde in different sampling occasions within treatment and control group. **p<0.01; ***p<0.001; TP1 - summer before treatment; TP2 - summer after treatment; TP3 - spring before treatment; TP4 - spring after treatment. TG - Treatment group; CG – Control group.

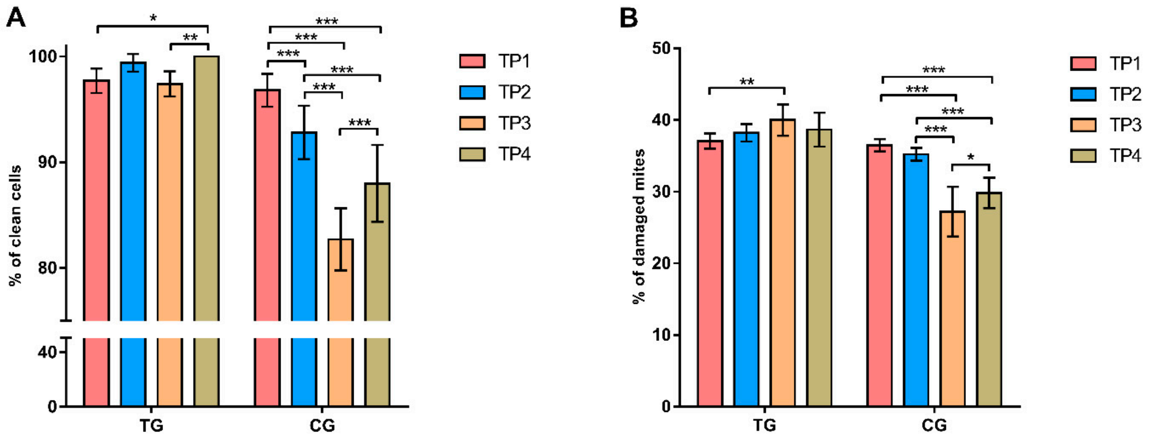

Supplementary Figure S4. Comparison of (**A**) hygienic and (**B**) grooming behavior in different occasions within treatment and control group. ***p*<0.01; ****p*<0.001; TP1 - summer before treatment; TP2 - summer after treatment; TP3 - spring before treatment; TP4 - spring after treatment. TG - Treatment group; CG – Control group.
